# Métastase oculaire révélant un carcinome canalaire du sein

**DOI:** 10.11604/pamj.2019.32.29.16466

**Published:** 2019-01-16

**Authors:** Saad Benchekroun Belabbes, Kawtar Belkhadir

**Affiliations:** 1 Service d’Ophtalmologie A Hôpital des Spécialités, Rabat, Maroc

**Keywords:** Métastase, sein, carcinome canalaire, Metastasis, breast, ductal carcinoma

## Abstract

We report the case of a 56-year old female patient, with no particular past medical history, presenting at the Emergency Department with decreased visual acuity in the right eye occurred 2 weeks before. Ophthalmologic examination showed reduced visual acuity to perception of hand movements. Fundus examination showed serous retinal detachment associated with subretinal mass occupying almost the entirety of the vitreous cavity (A). B-mode scan was performed which objectified isoechoic choroidal mass (B) with vascularization on Doppler (C) without any excavation, measuring 18 mm along its longer axis associated with serous detachment of the retina, highly suggestive of ocular metastasis. General assessment was performed and, in particular, mammography which revealed the presence of a nodule in the right breast (stage 4 according to BI-RADS Classification). Thoraco abdominopelvic and brain CT scan performed during staging evaluation showed many brain, bone and liver metastases. The diagnosis of metastatic breast cancer was made and the patient was referred to the Department of Oncology for specialist treatment. Ocular metastases are the most common intraocular tumors. Breast tumors are the main cause of choroidal metastases, justifying comprehensive assessment to highlight these intraocular tumors.

## Image en médecine

Nous rapportons le cas d'une patiente de 56 ans, sans antécédents pathologiques notables, ayant consulté aux urgences pour une baisse de l'acuité visuelle de l'œil droit, installée depuis 2 semaines. A l'examen ophtalmologique, on trouve une acuité visuelle réduite au mouvement de doigts, avec au fond d'œil un décollement de rétine séreux associé à une masse sous rétinienne comblant une majeure partie de la cavité vitréenne (A). Une échographie oculaire en mode B a été réalisée et a mis en évidence une masse choroïdienne isoéchogène (B) vascularisée prenant le doppler (C) sans excavation, mesurant 18 mm de grand axe associée un décollement séreux de la rétine, fortement évocatrice d'une métastase oculaire. Un bilan général a été réalisé et notamment une mammographie qui a révélé l'existence d'un nodule du sein droit stade 4 selon la classification BI RADS. Une Tomodensitométrie thoraco abdomino pelvienne, et cérébrale dans le cadre du bilan d'extension a montré la présence de nombreuses métastases cérébrale, osseuse et hépatique. Le diagnostic de cancer du sein métastatique a donc été posé, et la patiente a été référé en Oncologie pour prise en charge spécialisée. Les métastases oculaires sont les tumeurs intraoculaires les plus fréquentes. Les carcinomes mammaires représentent la principale cause des métastases choroïdiennes, justifiant un bilan exhaustif lors de la mise en évidence de ces tumeurs intra oculaires.

**Figure 1:**
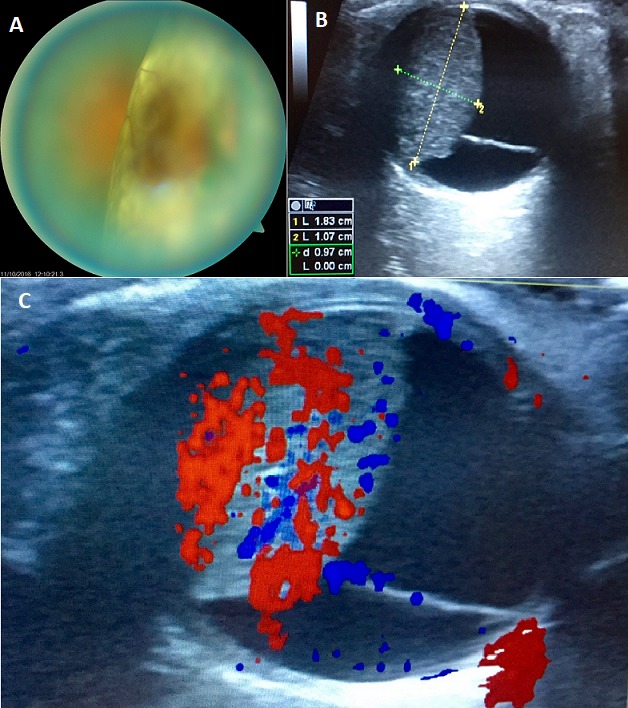
A) masse sous rétinienne occasionnant un décollement séreux; B) image échographique de la tumeur intraoculaire; C) vascularisation au Doppler de la tumeur intraoculaire

